# Identification of novel STAT3 inhibitors for liver fibrosis, using pharmacophore-based virtual screening, molecular docking, and biomolecular dynamics simulations

**DOI:** 10.1038/s41598-023-46193-x

**Published:** 2023-11-17

**Authors:** Huma Rafiq, Junjian Hu, Mohammed Ageeli Hakami, Ali Hazazi, Mubarak A. Alamri, Hind A. Alkhatabi, Arif Mahmood, Bader S. Alotaibi, Abdul Wadood, Xiaoyun Huang

**Affiliations:** 1 Department of Biochemistry, Computational Medicinal Chemistry Laboratory, Abdul Wali Khan University, Mardan, Pakistan; 2 Department of Central Laboratory, Dongguan Songshan Lake Central Hospital, Dongguan, China; 3 Department of Clinical Laboratory Sciences, College of Applied Medical Sciences, Al-Quwayiyah, Shaqra University, Riyadh, Saudi Arabia; 4 Department of Pathology and Laboratory Medicine, Security Forces Hospital Program, Riyadh, Saudi Arabia; 5 Department of Pharmaceutical Chemistry, College of Pharmacy, Prince Sattam Bin Abdulaziz University, 11942 Al-Kharj, Saudi Arabia; 6 Department of Biochemistry, College of Science, University of Jeddah, Jeddah, Saudi Arabia; 7 Center for Medical Genetics and Human Key Laboratory of Medical Genetics, School of Life Sciences, Central South University, Changsha, 410078 Hunan China; 8 Department of Neurology, Dongguan Songshan Lake Central Hospital, Dongguan, China

**Keywords:** Cancer, Computational biology and bioinformatics, Drug discovery

## Abstract

The signal transducer and activator of transcription 3 (STAT3) plays a fundamental role in the growth and regulation of cellular life. Activation and over-expression of STAT3 have been implicated in many cancers including solid blood tumors and other diseases such as liver fibrosis and rheumatoid arthritis. Therefore, STAT3 inhibitors are be coming a growing and interesting area of pharmacological research. Consequently, the aim of this study is to design novel inhibitors of STAT3-SH3 computationally for the reduction of liver fibrosis. Herein, we performed Pharmacophore-based virtual screening of databases including more than 19,481 commercially available compounds and in-house compounds. The hits obtained from virtual screening were further docked with the STAT3 receptor. The hits were further ranked on the basis of docking score and binding interaction with the active site of STAT3. ADMET properties of the screened compounds were calculated and filtered based on drug-likeness criteria. Finally, the top five drug-like hit compounds were selected and subjected to molecular dynamic simulation. The stability of each drug-like hit in complex with STAT3 was determined by computing their RMSD, RMSF, Rg, and DCCM analyses. Among all the compounds Sa32 revealed a good docking score, interactions, and stability during the entire simulation procedure. As compared to the Reference compound, the drug-like hit compound Sa32 showed good docking scores, interaction, stability, and binding energy. Therefore, we identified Sa32 as the best small molecule potent inhibitor for STAT3 that will be helpful in the future for the treatment of liver fibrosis.

## Introduction

Many studies have shown that signal transducers and activators of transcription 3 (STAT3) a crucial member of the STAT family of cytoplasmic proteins that transmit signals to the nucleus (in response to cytokines and growth factors) there by regulating growth, development, and survival of the cell^1^. STAT3 was identified as an acute-phase response factor that is linked with cancer and other diseases. In healthy cells, the STAT3 remains inactive, while it activates when the cell is experiencing unfavorable circumstances including cancer and other disease conditions. It is a transcription factor and is encoded in humans as the STAT3 gene. STAT3 consists of seven members (STAT 1, 2, 3, 4, 5A, 5B, and 6) encoded by different chromosomal clustered genes^2^. In addition to this Jiang-Jiang Qinet al suggested that STAT also consists of seven domains such as coiled-coil, SH2, NH2-terminus, DNA-binding, linker, and C-terminal Transactivation^3^. Among the STAT3 domains, the SH2 domain is the most significant. At Try705, C-terminal Transactivation activates STAT3 by generating serine and tyrosine development at position 727. The SH2 domains consist of 100 amino acidsthat control the tyrosine phosphorylation of STAT3 receptors and homo-dimerization or hetero-dimerization of STAT3 monomer bindings. Both are vital for gene expression as well as for DNA binding. While, SH2-, DBD, and N-terminal all have many functions, and are therefore considered therapeutic targets for many neurotic and pathological conditions^4^.

STAT3 is essential for cell development, growth, and regulation. Therefore, there are potential ways to inhibit STAT3 protein directly. On the basis of their structure and function the N-terminal, DNA binding domain and SH2 domain can be selectively targeted in order to design the STAT3 inhibitors. The SH2 components of the STAT3 are considered to play the most fundamental role in STAT3 activation. Among the different domains, the SH2 domain is preferentially targeted, because SH2 enables STAT3 to recruit it to any activated receptor. Furthermore, the inhibition of SH2 blocks STAT3 dimerization, thereby inhibiting nuclear translocation and STAT3-dependent gene regulation^5^.

TGFβ1, a STAT3 target gene, promotes liver fibrosis by increasing the expression of TGF-β1 in a liver fibrosis mouse model and a rapid early STAT3 activation was observed in HSCs and fibroblasts, but not in normal hepatocytes. This suggests that STAT3 could be a target for liver disorder. Moreover, STAT3 inhibition might reduce angiogenesis and further fibrogenesis thus, indicate STAT3 as a potential therapeutic target in liver disease^6,7^.

To overcome STAT3-induced diseases, several classes of direct low molecular weight inhibitors of STAT3 have been discovered that prevent STAT3-DNA or STAT3 dimerization binding. These inhibitors include oligonucleotides, peptides and peptidomimetics. However, it is challenging to develop highly potent and selective inhibitors of STAT3 against the liver fibrosis. In an attempt, computational screening precisely identified novel STAT3 SH2 inhibitors that block dimerization of STAT3, consisting of various chemical compounds such as STA-219, S3I-201 and STX-011911 which relied on docking of chemical libraries^1^. For chemical induced liver injury, some STAT3 inhibitors designed as chemical probes which are more affected in therapeutic sense. STX-0119 is reported as a more potent in controlling the improvement of CCl4 induced liver fibrosis via decrease the activated HSCs. Some other inhibitor like HJC0123was proven to overcome liver fibrosis by inhibiting the phosphorylation and block the process of STAT3 activation^8^.

Sorafenib is multi-kinase inhibitor and is considered as a potential inhibitor for liver fibrosis. Sorafenib has the potential to inhibit STAT3 phosphorylation^9^. It is also reported that Sorafenib has inhibited HSC activation which is also helpful in anti-fibrotic activity, growth and collagen accumulation in vitro.

Sorafenib plays a vital role in inhibiting STAT3 by directly binding to the SH2 domain that is SHP-1 is consisting of 2 Src homology domains, phosphorylated tyrosine binding, C-terminal tail and PTP catalytic domain. SHP-1 activity is linked closely with its structural changeability. SHP-1 is having auto inhibited ability but when the N-terminal SH2 domain on surface into the catalytic domain, blocking the active site of the catalytic pocket. The WPD loop containing the remaining active site residue Asp 421 was affected with this residue and the catalytic activity of SHP-1. The negative regulator of STAT3 is SHP-1. The anti-HCC effect of Sorafenib is due to the formation of important salts in the N-SH2 domain, which becomes the open catalytic PTP domain and liberates SHP-1. Sorafenib and its derivatives SC-1, SC-40 and SC-43 showed similar SHP-1 reactivation and STAT3 signal inhibition in HCC cells^10^.

In this study, Pharmacophore model of Sorafenib was generated using MOE software. The validation of Pharmacophore model was done by Gunner Henry method. Validated model was further used for virtual screening of different databases like in-house, Zinc, Phytochemicals and South African natural compounds database. In order to evaluate drug-likeness, screened hits were filtered with the help of SwissADME (http://www.swissadme.ch/) online server. After that hits were docked against STAT3-SH2. From docking study, top 05 complexes were selected for MD simulation. The selection criterion of the top 05 complexes for MD simulation was based on the S score and interactions with receptor. Finally, MD simulation analysis such as RMSF, RMSD, Rg, and DCCM shows that the new predicted inhibitors of STAT3 like Sa32, ZINC47009207, 5459840, and SANC00347 are more stable than control.

## Materials and methods

### Pharmacophore modeling and virtual screening

A Pharmacophore model was developed in the MOE (Molecular Operating Environment) software^11^ for the virtual screening of synthetic and commercially available compounds databases. The model was then validated and the validated Pharmacophore model as 3D query was utilized for the virtual screening. To identify and retrieve the potent anti-STAT3 compounds, different databases including in-house, ZINC, Phytochemicals, and South African databases were screened^12^. The screened hits best fitted on the applied Pharmacophore were further filtered by ADMET properties to short-list compounds having drug-likeness. In order to determine the pharmacokinetic properties of the retrieved compounds, the Lipinski’s rule of five (RoF) was applied. Determination of drug-likeness property is particularly used to demonstrate new lead hits by screening of compound databases^13^.

### Molecular docking of hit compounds and STAT3

The possible interaction of protein-ligands complex is explored by molecular docking study. Molecular docking helps topredict intermolecular framework formed among small molecule and protein, suggesting interaction modes leading to protein inhibition. In this study, all the drug-like retrieved hits were docked into the active site of STAT3. For docking purpose, the docking protocol implemented in MOE 2016 software was used^14^. For docking of STAT3, the structure of its SH2 domain (PDBID:1BG1) was taken from protein data bank https://www.rcsb.org/^15^ This SH2 domain (comprised of 499–688 amino acid sequence) of STAT3 is phosphorylated^16^. STAT3 receptor and its reference i.e., standard inhibitor (Sorafenib) was initially Protonated and subjected to energy minimization with default parameters (gradient: 0.05, Force Field: MMFF94x) of MOE 2016.08 software^17^. The energy converged system was subjected to docking. The result of docking was analyzed on the basis of protein/compounds interactions and docking scores. In order to validate the stability and interaction of drug-like hits in the binding site of STAT3-SH2 domain, the complexes were subjected to molecular dynamics simulations.

### Molecular dynamics simulations

MD simulation of the best five docking score compounds and the standard compound (Sorafenib) were carried out to unveil the stability of the protein–ligand complexes. Molecular dynamic simulations and analysis for each complex was performed in AMBER 20^18^ using the force field (*ff*1*4SB)*. During the MD protocol, each system was neutralized by utilizing the LEAP module counter ions such as Na^+^ and Cl^−^. TIP3P water model was filled in octahedral box with 10 Å parameter to solvateeach system^14^. For long-range electrostatic interactions a distance of 10 Å was used. Additionally, the Particle Mesh Ewald (PME) algorithm was used to deal with long-range electrostatic interactions. By using the SHAKE option and the Particle Mesh Ewald (PME) method, covalent bonds involving hydrogen atoms were optimized in each complex system^16^. The temperature was controlled by Langevin dynamics. Finally, 100 ns MD simulations (two replicas) for each complex were performed on the GPU using CUDA version of PMEMD. For the analyses of MD trajectory, Amber 20's CPPTRAJ module was used.

### ADME predictions

Evaluation of absorption, distribution, metabolism, and excretion (ADME) are the main criteria to recommend a molecule as a drug-like candidate. Hence, computational approaches are utilized to predict the ADME properties of chemical molecules and advocate their drug-likeness. In this study, we used SwissADME http://www.swissadme.ch online server^19,20^ and assessed the ADME properties such as GIT absorption, bioavailability, and solubility profile of the selected compounds^21^.

### Binding free energy calculation

The molecular Mechanic/Poisson-Boltzmann Surface Area (MM-PBSA) approach^22^ is an efficient and reliable method to model molecular recognition, such as for protein–ligand binding interactions. To estimate the strength of binding of STAT3 in complex with either the reference compound or any of the candidate hits, the binding free energy was computed by employing the MMPBSA.py script. The MMPBSA was computed for the last 500 frames (10 ns) of each simulated complex. The below equation was used to calculate the free energy^18^

$$ {\text{G}}\;{\text{bind}} = \Delta {\text{G}}\;{\text{complex}} - [\Delta {\text{G receptor}} + \Delta {\text{G ligand}}] $$

The equations describe *G bind* shows total binding free energies, *ΔG complex* use for complex free energies, and the remaining terms stand for the corresponding free energies of the receptor protein and ligand.

## Results

### Pharmacophore model generation

**Figure 1:**
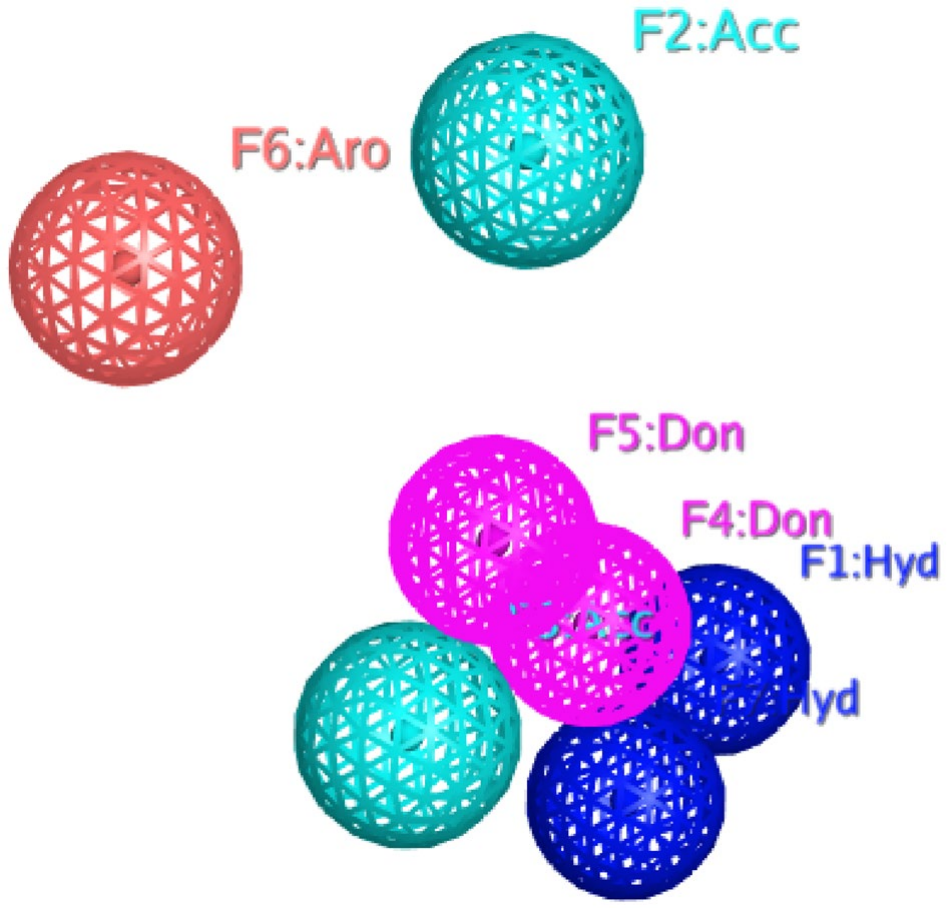
Represents features of validated Pharmacophore model which are represented with color-coded: Blue, one hydrophobic feature (F1 and F7: Hyd); Skyblue, Hydrogen bond acceptor features (F2 and F3: Acc); Pink, two hydrogen bond donor feature (F4 and F5: Don) Red, Aromatic feature (F6: Aro).

Ligand-based Pharmacophore model was generated. Sorafenib was selected as a reference for Pharmacophore model generation. Pharmacophorewas generated from a total of seven (7) features. Thus, the resulted Pharmacophore was comprised of two hydrogen bond acceptor (HBA), two hydrogen bond donor (HBD), one aromatic (Aro) and two hydrophobic (HY) features (Fig. 1). Finally, in-house, ZINC, phytochemicals and South African compound libraries were screened based on the generated Pharmacophore. As a result, many compounds were found that interacted more strongly with the targeted compound than the reference compound.

#### Pharmacophore model validation

Internal database was used in this study as test set forPharmacophore model validation. The test set contained 852 inactive compounds for STAT3 and the rest of 48 molecules were known inhibitors. The validated model has the ability to differentiate between the active and inactive molecules. This model was used to investigateand carry out virtual screening. Many important parameters were calculated like total hits (*Ht*), active hits (*Ha*), % yield of actives, % ratio of actives, enrichment factor (*E*), and goodness-of-hit score (*GH*) which are placed in (Table 1).

$$ \left( {Ha\,\left( {3A + Ht} \right)/4HtA} \right)\;\left( {1 - \left( {Ht - Ha} \right)/\left( {D - A} \right)} \right) $$

**Table 1:** Pharmacophore model evaluation based on Gunner-Henry (GH) scoring method.

S. no	Parameters	Ph4 model
1	Number of compounds in database (D)	900
2	Number of active in database (A)	48
3	Total Hits (Ht)	50
4	Active Hits (Ha)	40
5	% Yield of actives[(Ha/Ht) × 100]	80
6	% Ratio of actives [(Ha/A) × 100]	83
7	Enrichment factor (E) [(Ha × D)/(Ht × A)]	15
8	False negatives [A − Ha]	8
9	False positives [Ht − Ha]	10
10	GH score	0.79

GH Score of 0.7–0.8 indicates a validated model^23^. The model was used for further virtual screening purpose.

The higher the E value, the better the model's ability to identify active compounds. With an E-value of 15, the model identified 40 active hits out of 900 compounds screened, indicating that the model was able to differentiate active frominactive molecules. A *Gunner Henry’s* score of 0.7–0.8 preferred as a good model. So the goodness score for Pharmacophore model was 0.79. The validation of our generated Pharmacophore suggested that the resultant Pharmacophore was good enough to be used for virtual screening of different databases against the identification of STAT3 candidate hit compounds.

### Virtual screening

The validated model was utilized to screen in-house database (1700 molecules), zinc database (12,000 compounds), phytochemicals (5000 compounds) and South African database (1000 compounds). The Pharmacophore model retrieved340 compounds as the best fitted compounds on the Pharmacophore features against the STAT3 candidate hits molecules.
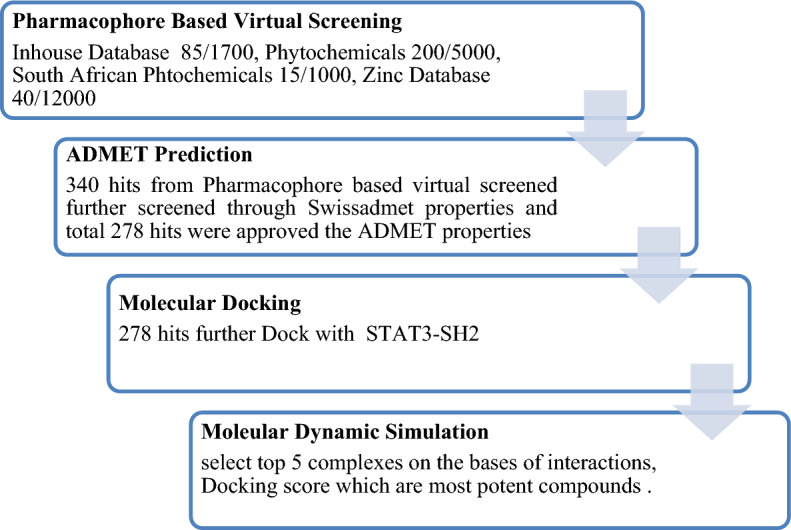


### Molecular docking

The ADMET evaluation of the Pharmacophore-retrieved hits filtered a total of 278 hits as drug-like molecules which were further docked in the active site ofSTAT3 which is comprised of Arg609, Arg595, Lys591, Arg609, Ser611, Glu612, Ser613, Gln633, Ile634, Gln635, Ser636, Val637, Gln638, Met586, Gly587, Phe588, Ilu589, and Ser590 residues. The top 25 ligands of STAT3 were selected according to the S-score and interaction type.

**Figure 2:**
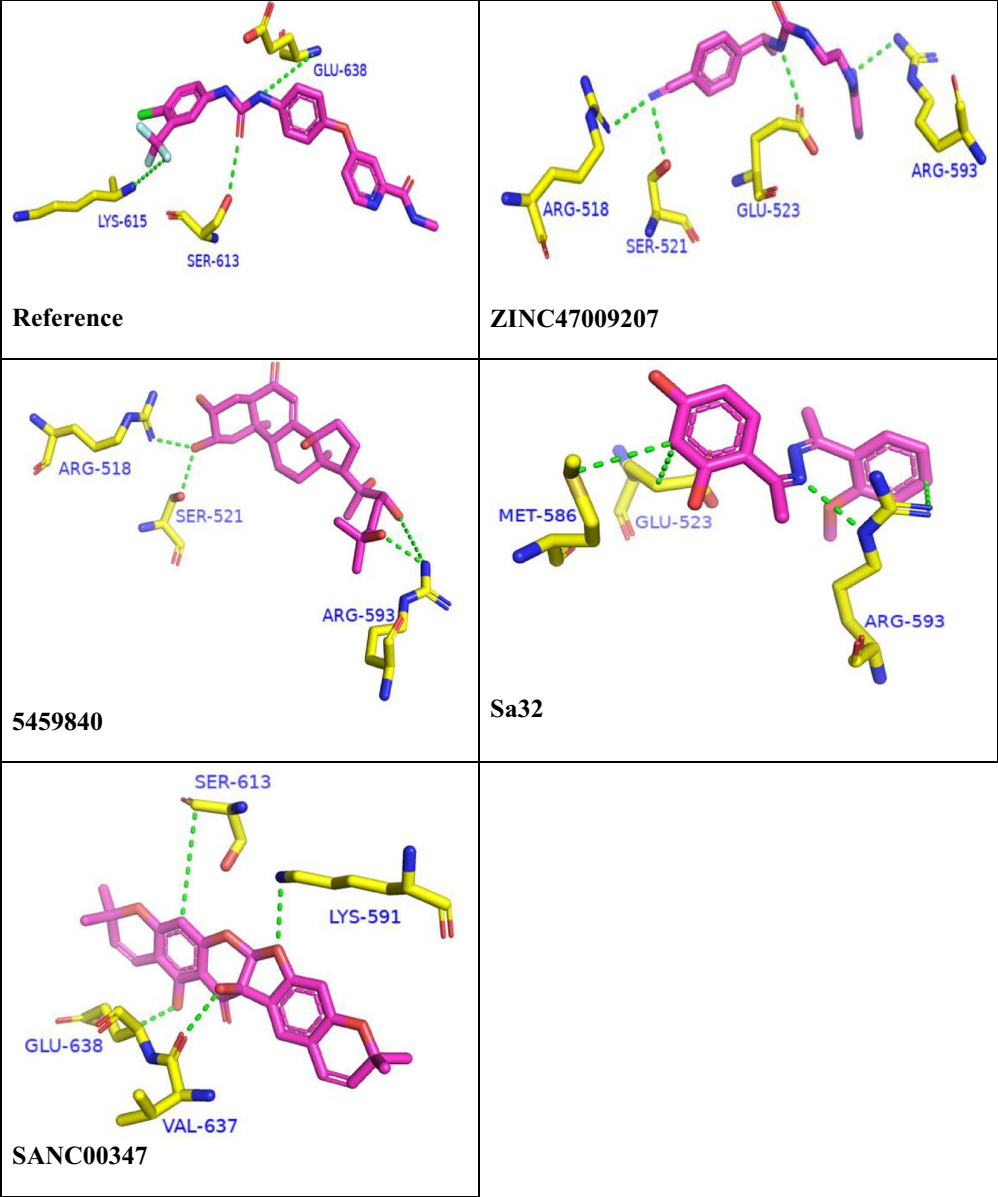
The binding interactions of compounds 1, 2, 3, 4 and 5. The interacting residues are depicting in blue color. The compounds are coded with pink color (stick model). Bonds are represented by green lines. The bond distances are labeled in green color. Compound’s names are written at bottom.

Table 2 suggests the S-Score and interacting residues of the top 25 hits with STAT3. Figure 2 represents the interactions of best hits and the reference compound with STAT3 receptor.

**Table 2:** The docking scores of top 25 ligands and their interactions with SH2 domain of STAT3.

S. no	Compound ID	Docking score	Interacting residues	Bond type	Distance
1	Ref	− 7.2334	GLU 638	H-donor	3.07
			SER 613	H-acceptor	3.18
			GLN 635	H-acceptor	3.53
2	5459840	− 11.2812	ARG 518	H-acceptor	2.85
			SER 521	H-acceptor	3.17
			ARG 518	H-acceptor	3.04
			ARG 593	H-acceptor	2.84
			ARG 518	Ionic	2.85
			ARG 518	ionic	3.98
3	SANC00347	− 10.2663	VAL 637	H-donor	2.89
			LYS 591	H-acceptor	3.15
			SER 613	pi-H	4.22
4	ZINC47009207	− 9.2654	GLU 523	H-donor	2.92
			ARG 518	H-acceptor	3.15
			SER 521	H-acceptor	3.17
			ARG 593	H-acceptor	3.29
5	sa32	− 9.1425	GLU 523	H-donor	3.71
			ARG 593	H-donor	4.38
			ARG 593	H-acceptor	3.01
			MET 586	Pi-H	3.1
6	SANC00352	− 10.1882	LYS 591	H-acceptor	3.48
			SER 636	H-acceptor	3.23
			GLU 638	pi-H	4.29
7	SANC00512	− 9.9356	GLU 594	H-donor	2.80
			GLN 635	H-acceptor	3.21
			LYS 626	H-acceptor	3.21
8	442659	− 9.4941	GLU 523	H-donor	3.58
			ARG 593	H-acceptor	2.97
			TYR 674	Ionic	2.92
9	10885340	− 9.7567	GLU 612	H-donor	3.12
			LYS 591	H-acceptor	2.86
			SER 613	H-acceptor	2.99
10	ZINC44978511	− 9.1258	MET 586	H-donor	4.17
			GLU 530	H-donor	3.07
			ARG 593	H-acceptor	3.30
11	ZINC23543539	− 9.0425	GLU 523	H-donor	2.83
			GLU 530	H-donor	3.28
			ARG 593	H-acceptor	2.93
12	SANC00856	− 9.4064	GLU 594	H-donor	2.79
			LYS 591	H-acceptor	3.05
			SER 613	H-acceptor	3.03
13	SANC01083	− 8.2785	ILE 634	H-donor	3.01
			GLU 594	H-donor	2.91
			SER 636	H-acceptor	3.12
14	6-KA6-TP	− 8.1504	MET 586	H-donor	3.81
			GLU 523	H-donor	2.84
			GLU 523	pi-H	3.73
15	ZINC48237730	− 8.3923	MET 586	H-donor	3.78
			MET 586	H-donor	3.75
			GLU 523	pi-H	4.36
16	16736655	− 8.1621	GLU 523	H-donor	3.36
			ARG 593	H-acceptor ionic	3.17
			GLU 523	H-acceptor ionic	3.81
17	9957384	− 7.9068	ARG 595	H-acceptor	2.90
			LYS 591	Ionic	2.86
			SER 636	pi-H	4.32
18	ZINC20540819	− 7.8776	MET 586	H-donor	3.99
			GLU 523	pi-H	3.76
			MET 586	pi-H	4.60
19	7-KA7-TP	− 7.8152	MET 586	H-donor	3.79
			GLU 523	H-donor	2.83
			GLU 523	pi-H	3.72
20	8.3-Formylcoumarin	− 7.7606	GLU 594	H-donor	2.77
			GLU 594	H-donor	2.89
			GLU 638	H-acceptor	3.21
21	BB-III-18	− 7.7005	GLU 523	H-donor	2.98
			ARG 593	H-acceptor	3.10
			TYR 674	pi-H	3.92
22	Sa5	− 8.6211	GLU 594	H-donor	2.89
			ILE 634	Pi-H	4.78
			SER 636	Pi-H	4.38
23	Sa10	− 7.7093	MET 586	H-donor	3.51
			GLU 523	H-donor	3.64
			TYR 674	Pi-H	3.51
24	Sa12	− 9.7339	MET 586	H-donor	3.97
			TYR 674	H-pi	4.26
			GLU 523	Pi-H	3.70
25	Sa15	− 8.06696	TYR 674	H-pi	4.23
			MET 586	Pi-H	4.15
			PRO 675	Pi-H	3.94

Among the 25 docked compounds, 5459840, SANC00347, ZINC47009207 and Sa32 were the most potent with docking score  − 11.2812, − 10.2663, − 9.2654 and − 9.1425, respectively. Most of the compounds showed the most accurate binding and interaction with the receptors compared to reference compound. The docking index for Sorafenib was − 7.2334.

### ADMET properties determination

**Figure 3:**
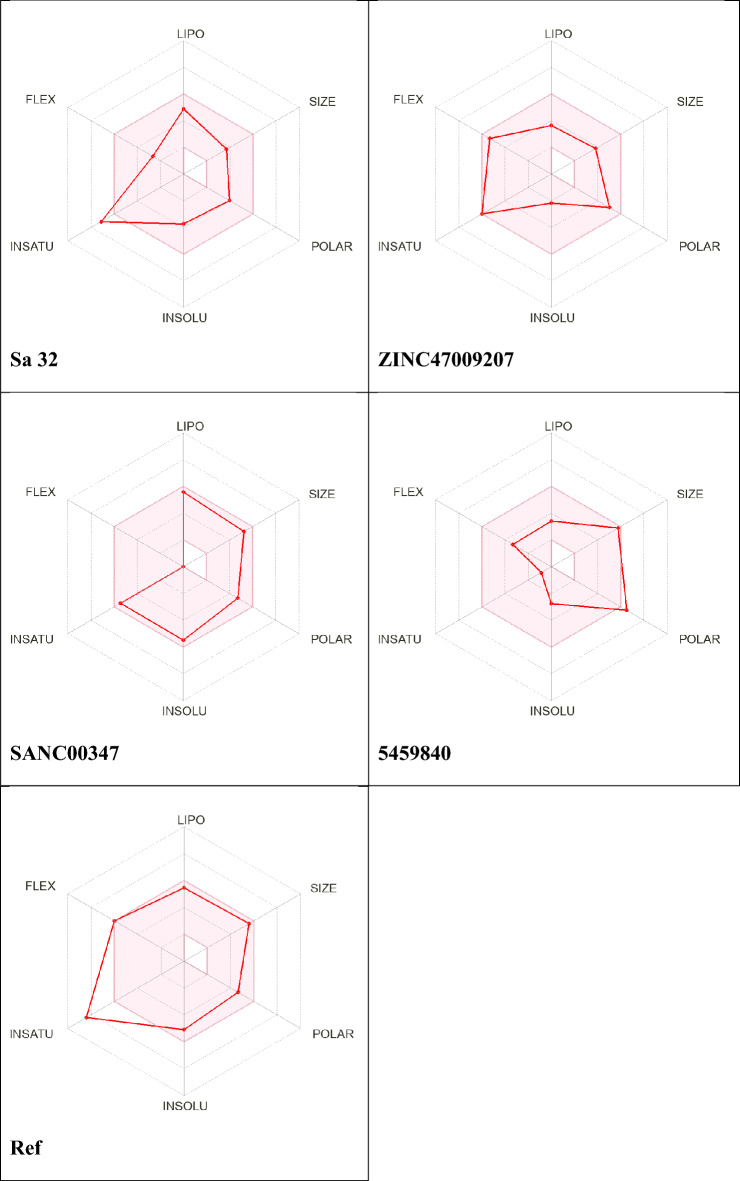
SwissADME radar of various bioactive drug likeness molecules, the red areas in discern constitute every attribute of lipophilicity, solubility, molecular weight, and versatility.

Table 3 shows the drug likeness of the molecules 5459840; ZINC47009207, Sa32 and SANC00347 identified by online ADME/Tox tool of Swiss-ADME using the SMILES nomenclature. To show the ADME/Tox profile, we decided on maximum fundamental ADME/Tox functionality from online server. Table 3 displays the pharmacokinetic profiles of the selected compounds. Drug-likeness of a compound is strongly stimulated with the aid of 2 properties of water solubility and hydrophobicity (predicted using the ALI log S, log S, and ESOL methods)^24^. Figure 3 shows the drug-like properties (molecular weight etc.) obtained from ADME/Tox tool. Orally active drugs must follow Lipinski rules five.

**Table 3:** Drug likeness properties of the nominated compounds.

S. No	Compounds ID	MW	TPSA	HB donor	HB accep	No. Rot bond	LogP	LogS(ESOL)	LogS(Ali)	Violation
1	Ref	464	92.35	3	7	9	4	− 5.11	− 5.71	0
2	5459840	478	144.11	4	7	5	1.94	− 2.77	− 3.05	0
3	SANC00347	434	94.45	2	7	0	3.46	− 5.49	− 5.96	0
4	ZINC47009207	310	102	3	4	8	1.06	− 2.16	− 2.60	0
5	sa32	298	74	2	5	4	3.02	− 3.72	− 4.23	0
6	SANC00352	436	109	3	7	1	3.51	− 5.34	− 5.95	0
7	SANC00512	664	151	4	7	10	3.67	− 6.00	− 7.20	0
8	442659	372	76.36	0	7	6	3.10	− 4.40	− 4.79	0
9	10885340	481	157	4	10	4	1.64	− 4.14	− 4.84	0
10	ZINC44978511	296	82.07	2	3	2	1.19	− 2.28	− 1.71	0
11	ZINC23543539	311	136	2	6	9	0.89	− 2.00	− 3.18	0
12	SANC00856	354	100	3	6	2	2.62	− 4.27	− 4.80	0
13	SANC01083	436	188	4	10	6	− 0.22	− 2.54	− 3.68	0
14	6-KA6-TP	321	83.81	2	5	5	2.47	− 3.86	− 4.38	0
15	ZINC48237730	308	83.16	0	7	3	1.89	− 2.94	− 2.68	0
16	16736655	436	53.35	1	3	5	1.19	− 3.93	− 3.04	0
17	9957384	383	66.46	0	7	3	2.57	− 4.06	− 3.79	0
18	ZINC20540819	258	85	1	5	5	1.22	− 2.22	− 2.21	0
19	7-KA7-TP	309	54.35	1	3	4	3.40	− 4.54	− 4.81	0
20	8.3-Formylcouma	374	132	4	7	4	1.96	− 3.81	− 4.73	0
21	BB-III-18	369	87	2	4	3	3.21	− 5.07	− 5.72	0
22	Sa5	325	68	2	4	6	3.19	3.27	− 4.23	0
23	Sa10	284	74	2	4	4	2.54	2.60	− 3.52	0
24	Sa12	344	92	2	7	6	2.40	2.45	− 3.70	0
25	Sa15	314	83.64	2	6	5	2.44	2.50	− 3.59	0

### Molecular dynamic simulation

**Figure 4:**
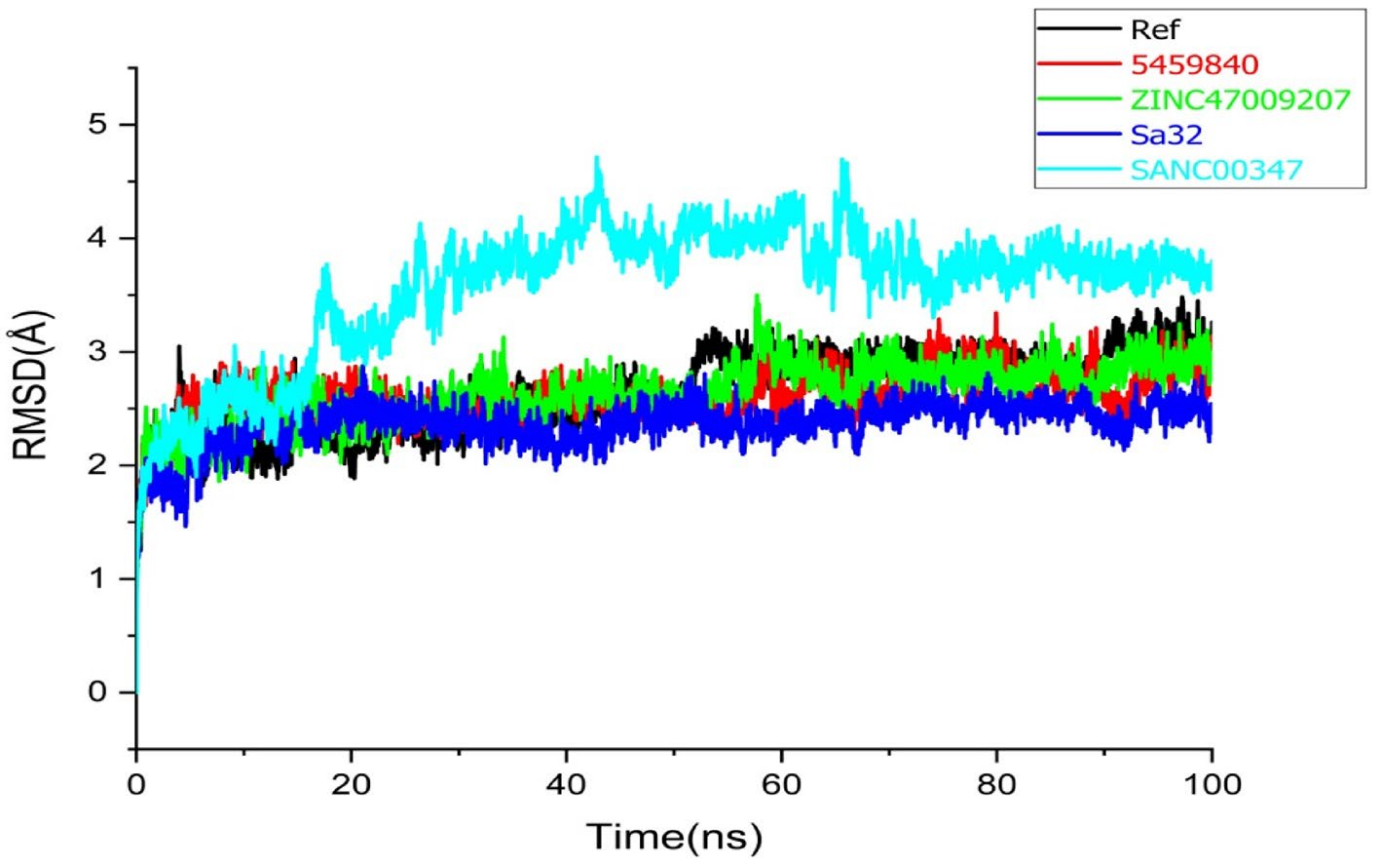
Root-mean square deviation (RMSD) analysis, black plot in graph shows the reference complex and Red, Green, Blue and Sky blue plotted indicates 5459840, ZINC47009207, Sa32 and SANC00347 (replica 1) respectively.

**Figure 5:**
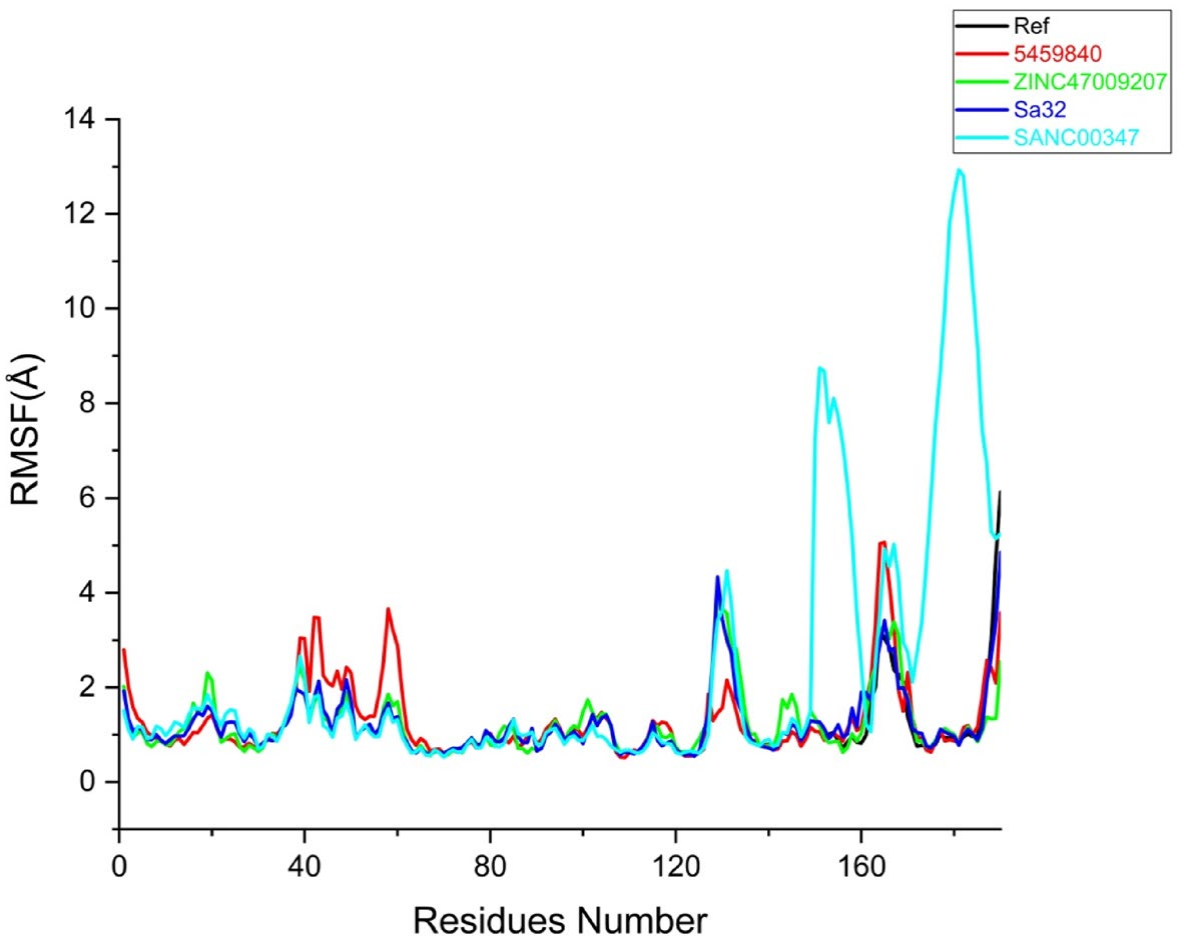
Root-mean-square fluctuation analysis the black line in graph represents reference complex. And the rest of Red, Green, Blue and Sky blue indicate 5459840, ZINC47009207, Sa32 and SANC00347 (replica 1) respectively.

**Figure 6:**
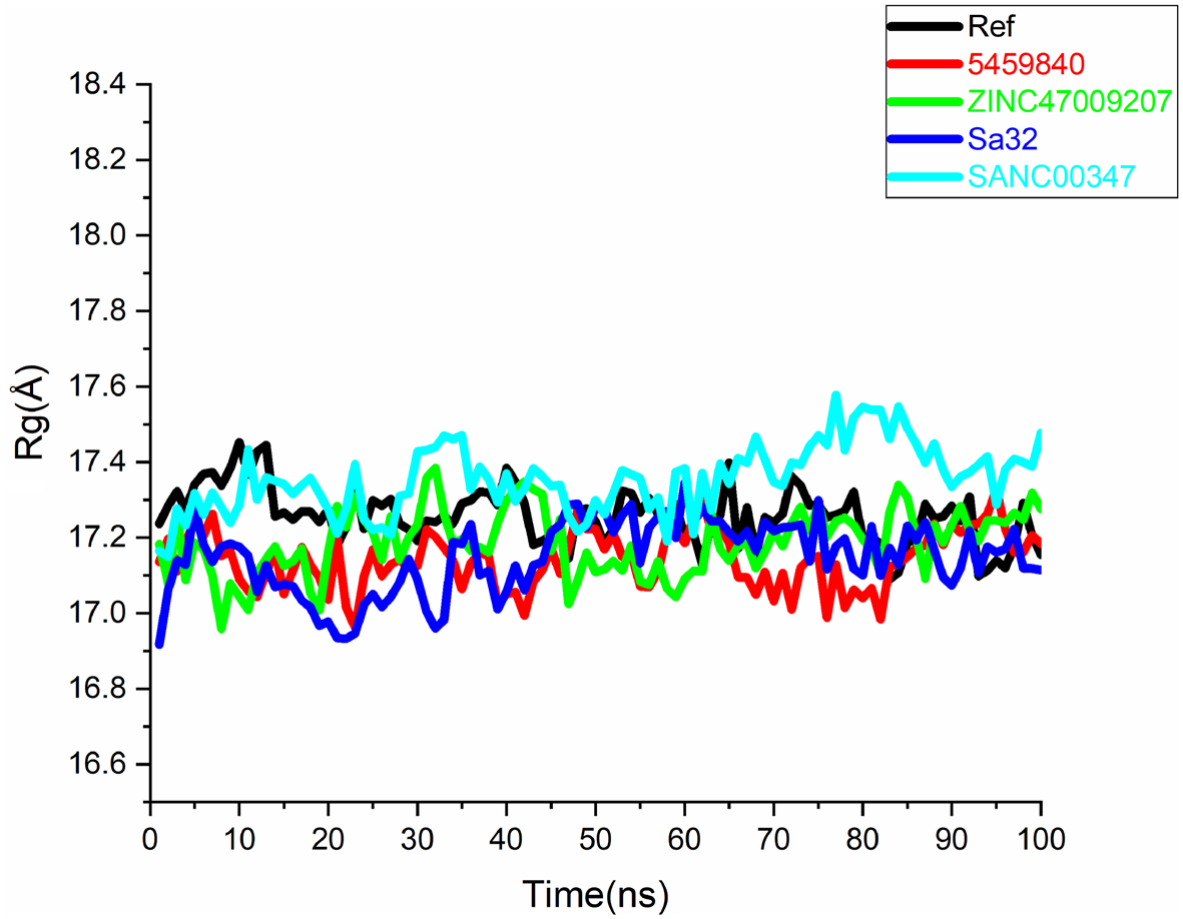
Rg analysis of replica 1, Black, Red, green, blue and sky-blue shows Ref, 5459840, ZINC47009207, Sa32 and SANC00347 respectively.

**Figure 7:**
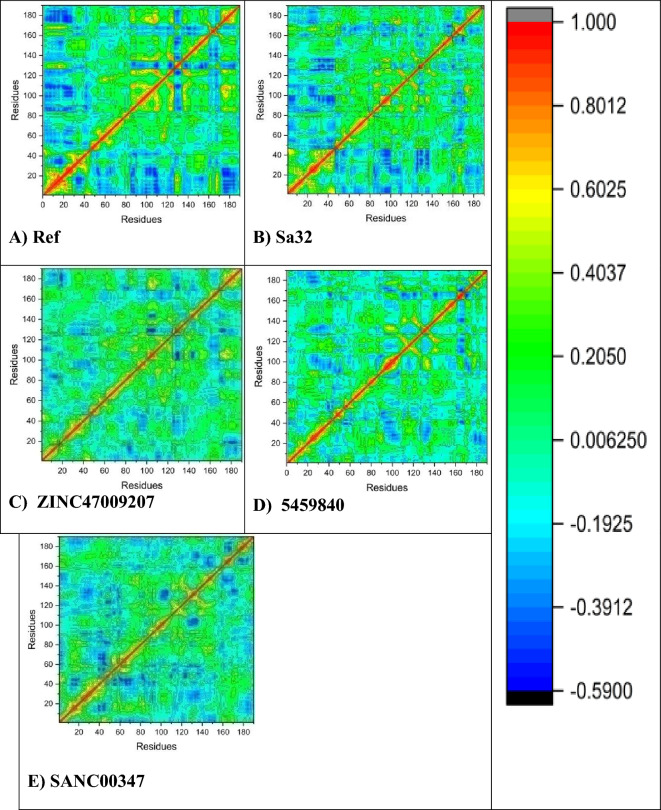
DCCM comparative analysis of natural and synthetic compounds (**A**) DCCM of the reference compound, (**B**) Sa32 (**C**) ZINC47009207 (**D**) 5459840, and (**E**) DCCM of SANC00347.

To investigate the structural stability of the docked complexes, 100 ns molecular dynamics simulations were executed to determine the stability of the top lead compounds in complex with receptor. MD simulations were performed by using Amber program software. Molecular dynamics (MD) simulations of the top five (05) hits with good interactions, binding energies, best docking scores, and have satisfied ADMET properties compared to the reference molecule was carried out. While, stability of the complexes ZINC47009207 (green) compound, Sa32 complex (Blue), and SANC00347 (sky-blue) were evaluate based on root mean square deviation (RMSD), root mean square fluctuation (RMSF), radius of gyration (Rg) and DCCM. Moreover, to recognize the structural variability in protein over a time of 100 ns, the simulated complex with existence of lead compounds at particular trajectory frames that is frame 1, 20, 40, 60, 80, and 100 were analyzed and are shown in Figs. 4, 5, 6 and 7.

#### RMSD analysis

The predicted complexes stability was further checkedby MD simulations. After 100 ns MD simulations, the RMSD of the backbone of STAT3 and the ligands at 300 K was plotted against time (ns). This was shown in Fig. 4, the RMSDs of Sa32 and ZINC47009207 were found to be comparatively firm and stable at around 2.7 and 3 Å, respectively. Sa32 wasstable from the beginning in replica 1 then there were some fluctuations from 40 to 60 ns. The Sa32 in replica2 revealed minor deviations from 5–10 to 70–85 ns but overall the complex RMSD was stable during the 100 ns MD run.ZINC47009207 in replica 1has been stable since beginning with some unstable behavior from the 60 ns to 70 ns as compared to Ref complex. The ZINC47009207in replica2 revealed an unstable behavior during 21–70 ns but then the system gain stability and remained stable till the end of MD run. The average RMSD value of the reference compound was found as 3.1 Å in replica 1. The RMSD of Ref compound in replica2 was stable during the first 25 ns and then the RMSD increases from 26 ns to 50 ns after that the system gain stability and remained stable till the 100 ns. The compound545984 in replica 1around 20 ns show fluctuation then get stable and afterward from 50 ns to 65 unstable and from 85 to 90 ns show fluctuation. The RMSD value of the 545984 was found as 2.8 Å during simulation. The RMSD of compound 545984 in replica2 indicate stable during the first 50 ns after that a slight increase in RMSD was observed and fluctuations were observed during 51–82 ns after that, the system gain stability and remained stable. The compound SANC00347 shows stability from start to 20 ns then gradually shows fluctuation from 21 to 80nsin replica 1. Then from 85 to 100 ns they show stability. The RMSD value of SANC00347 was found as 4.5 Å during MD simulation. The RMSD of SANC00347 in replica2 indicates a similar pattern to that of the replica1. The RMSD was stable during the first 20 ns but major deviations were observed from 20 to70 ns and then get stability and remained stable during the last 30 ns. The RMSD plots of replica 2 are shown in Fig. S4 (Supplementary). Except the compound SANC00347 the RMSD of all other compounds indicated high stability during the entire 100 ns MD simulation.

#### RMSF analysis

The RMSF is a significant factor that produces data regarding the structural flexibility of each and every residue i-e remains in the system. In replica 1 the RMSF values of the Ref, 5459840, ZINC47009207, Sa32 and SANC00347 complexes were calculated (Fig. 5). In RMSF we found that fluctuation was found in the region including 18–21(THR516, LYS517, ARG518, GLY519) 39–41(VAL537, ASN538, TYR539) 43–51(SER540, GLY541, CYS542, GLN543, ILE544, THR545, TRP546, ALA547, LY548, PHE549, CYS550, LYS551) 122–127 (THR620, PHE621, THR622, TRP623, VAL624, GLU625) 160–164(LYS658, ILE659, MET660, ASP661, ALA662). The fluctuating residues were not present in the binding site and the active site residues showed stable behavior which indicates strong binding of compounds with the receptor. The RMSF value of SANC00347 was very high up to 12 Å while the RMSF value of all other complexes was less than 4 Å. RMSD analysis was in agreement with the RMSF analysis which indicated that among all the complexes SANC00347 was unstable as compared to the control and other complexes. In replica 2 a similar pattern of RMSF was observed as the replica 1. In all the systems the RMSF was found less than 4 Å except the SANC00347. The RMSF plot of SANC00347 was observed as 13 Å. The residues such as 25–40 and 125–135 in the complexes 5459840 and the SANC00347 revealed high fluctuations during the MD simulation. The residues of Sa32 and ZINC47009207 revealed minor fluctuations during MD simulation. The RMSF plots of all the systems in replica 2 are shown in Fig. S5 (Supplementary).

#### Radius of gyration (Rg)

STAT3 protein compactness with their complexes was evaluated by utilizing the radius of gyration (Rg). The analysis of Rg confirmed that all complexes of STAT3 i.e., Sa32 and ZINC47009207 while Fig. 6 showed a divergent pattern of compactness. Extensively, the Rg of the Sa32 and ZINC47009207 complexes show stability throughout MD simulation and demonstrate a vigorous compact conformation. The Rg value of SA32 (replica 1) was found as 16.9–17.3 Å. The Rg value of ZINC47009207 (replica 1) was found as 17.2–17.4 Å. The Rg value of SANC00347 (replica 1) was 17.2–17.5 Å. The Rg value of 5459840 (replica 1), and Ref (replica 1) were found to be 17.1–17.3 Å and 17.2–17.41 Å respectively. Whereas, other 5459840 and SANC00347 complexes were indicated to be less compact over time as compared to Ref. The dynamic behavior of protein–ligand complexes reveal that binding alters stability and remaining flexibility, thereby inducing therapeutic ability. In replica 2 the Rg of the SANC00347 was observed to be 17.1–17.5 Å. The Rg of the complex 5459840 was observed to be 17.0–17.4 Å. The Rg value of ZINC47009207, Sa32 and Ref was found to be 16.9–17.1, 16.6–17.1 Å and 16.4–17.1 Å respectively. The Rg plots of replica 2 of all the complexes are shown in Fig. S6 (Supplementary). As compared to replica 2 all the complexes were more compact and revealed high stability in replica 1.

#### DCCM analysis

The aim of the DCCM analysis is to study the changes in the binding pocket conformation of STAT3 protein when interacting with various ligands. This was accomplished through a post Molecular Docking analysis of the backbone of Ca during MD simulations lasting 100 ns, to observe any fluctuations and correlated motions. To determine binding affinity of selected or targeted ligands in the active site of the correlation and anti-correlation motions of STAT3. The dynamics matrix of cross-correlation plots of 100 ns MD simulations was plotted for every complex. The analysis of DCCM was to evaluate correlation between residuals and this was performed to elucidate the motion correlation of the residuals. Movements of amino acids are in parallel direction, indicating strongly correlated movements called positive correlation. On the other side, if the amino acids movements are anti-parallel then this shows movement of anti-correlation. Negative & positive correlations were calculated by DCCM analysis and represented as the cyan to dark blue region and the red to green region as shown in Fig. 7A–E

### Free energy binding

The binding energy of ligand-STAT3 complexes was calculated using MMPBSA methods. However, van der Waals and electrostatic interactions and non-polar solvation energy negatively contribute to the total interaction energy while only polar solvation energy positively contributes to total free binding energy. In terms of negative contribution, van der Waals interaction gives much larger contribution than electrostatic interactions for all the cases. The non-polar free energy contributes relatively less as compared to the total binding energy. This indicates that non-polar solvation energy, van der Waals and electrostatic interaction together contribute to the STAT3 polyphenol complex stability^25^. MMPBSA was calculated for the last 10 ns from the total of 100 ns MD trajectory. The binding free energies ΔG Bind of complexes Ref, 5459840, ZINC47009207, Sa32 and SANC00347 calculated during the 100 ns simulation were found to be − 9.8027 kcal/mole, − 11.5849 kcal/mole, − 10.6547 kcal/mole and − 24.0920 kcal/mole respectively. The details of MMPBSA calculation of the complexes are summarized in Table 4. In Table 4 shown the calculated values of the molecular mechanic’s VDW, EFL, EPB, ENPOLAR and EDIPER under ΔPBSA between the protein and ligand during the simulation were evaluated from the MM-PBSA calculation. The van der Waals VDW interactions of ligands − 29.1030 kcal/mole (Reference), − 29.6634 kcal/mole (5459840), − 43.4243 kcal/mole (ZINC47009207), − 34.0802 kcal/mole (Sa32) and − 35.0421 kcal/mole (SANC00347), all the complex systems showed the negative values of VDWso all complexes illustrated sufficient hydrophobic interactions. The results of the free energy calculation showed that the complex having best binding energy, also best RMSD and best binding affinity toward receptor. As compared to Ref compound binding energy of SANC00347 was not good enough.

**Table 4:** MMPBSA free binding energy of selected compounds.

S. No	Complex	VDW	EEL	EPB	ENPOLAR	EDIPER	ΔPBSA
1	Reference	− 29.1030	− 1.2460	15.3688	− 17.9212	34.7609	− 9.8027
2	5459840	− 29.6634	39.2518	− 11.9422	− 18.7723	32.7110	− 11.5849
3	ZINC47009207	− 43.4243	− 2.5194	16.7161	− 23.7484	43.3213	− 10.6547
4	Sa32	− 34.0802	− 140.0321	132.3842	− 24.8220	42.4582	− 24.0920
5	SANC00347	− 35.0421	− 3.3731	15.7086	− 21.1103	37.0142	− 7.8595

## Discussion

Liver fibrosis is an initial stage of cirrhosis and results from abundant chronic liver disorders and a wide process that presents with numerous depositions of extracellular matrix proteins that change the liver architecture and functionality resulting in cirrhosis and failed organ^26^. Liver disorders are responsible for approximately 2million fatalities each year worldwide, with 1million of these deaths attributable to cirrhosis severity. Liver fibrosis is now at the top of list among ten causes of death globally^27^. Therefore, some STAT3 inhibitors exist such as Sorafenib inhibits STAT3 phosphorylation in human tumors, HCC, and liver fibrosis etc. However, STAT3 played a beneficial role against liver fibrosis due to the proliferative function of STAT3.

STX-0119 is a STAT3 inhibitor of dimerization. STX-0119 mediates the development of CCl4 and thioacetamide- induced liver fibrosis by diminishing the activation of HSCs^28^.

There are some STAT3 SH2 domain inhibitors that are Shikonin a natural naphthoquinone derivative that has been recognized as STAT3 inhibitory potency. Napabucasin has been also identified as a STAT3 inhibitor which inhibited STAT3 by binding to its SH2 domain. Nifuroxazide and ODZ10117 have a greater inhibition on STAT3 activation via directly blocking the SH2 domain of STAT3 and blocking the transcription activity^29^.

This study has shown few STAT3 inhibitors that help to overcome STAT3 activity as well as anticancer effects. According to Tomohiro, Von Manstein, and Groner there are also some disadvantages, such as dose-limiting side effects, multidrug resistance, and extreme side effects. The result is a timetable for the development of essentially new STAT3 inhibitors with improved viability^30^.

The overall analysis of novel STAT3 inhibitors from different Databases such as In-house (Sa32), Zinc(ZINC47009207), Phytochemicals(5459840) and South African (SANC00347) were carried out. The docking interaction of compounds, RMSD, RMSF, DCCM, Rg, and MMPBSA based analysis are discussed below.

From previous study of docking results shows that N4 (inhibitor) which are STAT3 inhibitor interacted with STAT3-SH2 at multiple amino acid residues some are following LYS591, SER636, VAL637, AND GLU638^31^ STAT3 inhibitor i-e S31-201with compounds which improved bioavailability and anti-cancer properties. Their mechanism of action depends upon interaction with STAT3-SH2 and phosphor tyrosine 705 site. The paining with active dimmer from tyrosine peptide is represented together with inhibitor S31-201 from H-bind and residues including LYS591, SER611, SER613 and ARG609^32^.

In current study Sorafenib dock as a reference drug of STAT3 and Sorafenib interact with STAT3-SH2 with some common residues same as previous study that is shown in Fig. 2 they also show structures similarity and binding interaction. And South African compounds SANC00347 show some similarity with S31-201 inhibitor with STAT3-SH2 interactions as shown in Fig. 2 S31-201 and SANC00347 show interaction on same active site residue.

RMSD provides detailed information about protein and their structure predictions. In previous studies RMSD of STAT3 inhibitor (−)-Epigallocatechingallate-complex, Saikosaponin D complex, Kaempferol-3-*O*-rutinoside complexes and Ginsenoside RK1 and Picroside. On the other hand, the bound state of the protein can stabilize between 50 and 100 ns. For the complex the RMSD was found to exceed 1.5 nm, with multiple variations for Ginsenoside RK1 and a decrease in stability for Picroside I within 75 ns. Both compounds showed lower stability and higher RMSD due to multiple amplitudes. A number of STAT3 complexes are observed by evaluating the active behavior of backbone residues and atomic position variations^24^. In the current study RMSD analysis of novel STAT3 inhibitors such as Sa32, ZINC47009207, 545984 and SANC00347 complexes show stability but also fluctuate at some positions as compared to Reference. But Sa32 and ZINC47009207 best RMSD and binding affinity shows in Fig. 4.

The previous RMSF result provides valuable information about the dynamic behavior of the residues. A reduced flexibility of the complexes will occur due to a small change in atomic positional Hicks^24^.

In the current study, RMSF is the fundamental factor that provides data on the structural compliance of each residue in the system. The values of the following that is Ref, 5459840, ZINC47009207, Sa32 and SANC00347 complexes were calculated Fig. 5 in RMSF we found that fluctuation was found in the region including 18–21, 39–41, 43–51, 122–127 and 160–164 respectively. Some hikes present which are not active site residues.

The current STAT3 inhibitors which are analyzed by Rg analysis and as an important residue amplitudes and backbone deviation, more comprehensive check, general compression in all complexes were needed. The analysis of Rg shows that all complexes of STAT3 i.e., Sa32 and ZINC47009207, 5459840 and SANC00347 showed a different pattern of compared to Ref, as shown in Fig. 6. The dynamic behavior of protein–ligand complexes reveal that binding alters stability and residual flexibility, there by inducing therapeutic clouds.

To investigate the structural changes in the binding pockets of STAT3 protein, to determine the interacting effects of selected compounds in the active site of STAT3 on positively correlated and negatively correlated movements. Hence, for each complex system, the dynamic cross-plots of the correlation matrix are plotted for 100 ns MD simulation, which analyzed the regions of negative and positive correlation, such as cyan to dark blue region and red to green, as shown in Fig. 7A–E.

MMPBSA Binding Free Energy (Kcal/mol) is calculated for nominated complexes for estimated protein–ligand complexesof 100 ns MD trajectory. The overall results of complexes were compared and on the basis of MMPBSA analysisSa32 hadthe best binding energy which is shown in Table 4. Sa32 is also best in all analyses like RMSD, and Rg indicating the strong binding affinity of the Sa32 compound toward the receptor.

## Conclusion

In the current study, novel STAT3 inhibitors were identified with a comprehensive screening method. The Ph4-based virtual screening, docking, and MD simulations were carried out. Additionally, 25 complexes were selected based onbest docking scores which were further screened for the drug-likeness. ADMET drug similarity was utilized to filter the properties of pharmacokinetic of the designed ligand–protein complexes. Lastly, 5 molecules were selected having excellent properties as well as stable binding modes. In addition, Sa32 has a valuable and novel STAT3 inhibitor, which will further serve as a reference for the development of new effective STAT3 inhibitors. Sa32 compound is overall showing the best response toward STAT3 inhibition which is confirmed through RMSD, RMSF, RG and DCCM analysis. It is further recommended to perform *Invitro* study on the final predicted hits to evaluate the inhibitory potential of these compounds.

### Supplementary Information

Supplementary Information.

## Data Availability

All the data and its links are available in the manuscript.

## References

[1] Poli G (2016). Identification of a new STAT3 dimerization inhibitor through a pharmacophore-based virtual screening approach. J. Enzyme Inhib. Med. Chem..

[2] Tolomeo M, Cascio A (2021). The multifaced role of STAT3 in cancer and its implication for anticancer therapy. Int. J. Mol. Sci..

[3] Qin J-J (2019). STAT3 as a potential therapeutic target in triple negative breast cancer: A systematic review. J. Exp. Clin. Cancer Res..

[4] Lakshmanan K (2022). Discovery of potential inhibitors for stat3: Ligand based 3D pharmacophore, virtual screening, molecular docking, dynamic studies and in vitro evaluation. J. Biomol. Struct. Dyn..

[5] Wang X (2012). STAT3 inhibition, a novel approach to enhancing targeted therapy in human cancers. Int. J. Oncol..

[6] Wang Z, Long J, Zhang H (2018). The STAT3 inhibitor S3I–201 suppresses fibrogenesis and angiogenesis in liver fibrosis. Lab. Invest..

[7] Tang M (2021). Therapeutic targeting of STAT3 with small interference RNAs and antisense oligonucleotides embedded exosomes in liver fibrosis. FASEB J..

[8] Alkreathy HM, Esmat A (2022). Lycorine ameliorates thioacetamide-induced hepatic fibrosis in rats: Emphasis on antioxidant, anti-inflammatory, and STAT3 inhibition effects. Pharmaceuticals.

[9] Deng Y-R (2013). STAT3-mediated attenuation of CCl4-induced mouse liver fibrosis by the protein kinase inhibitor sorafenib. J. Autoimmun..

[10] Hung M-H (2014). Downregulation of signal transducer and activator of transcription 3 by sorafenib: A novel mechanism for hepatocellular carcinoma therapy. World J. Gastroenterol. WJG.

[11] Wadood A (2022). Machine learning-based virtual screening for STAT3 anticancer drug target. Curr. Pharm. Des..

[12] Irwin JJ (2012). ZINC: A free tool to discover chemistry for biology. J. Chem. Inf. Model..

[13] Muegge I (2003). Selection criteria for drug-like compounds. Med. Res. Rev..

[14] Wadood A (2021). In silico drug designing for ala438 deleted ribosomal protein S1 (RpsA) on the basis of the active compound Zrl 15. ACS Omega.

[15] Becker S, Groner B, Müller CW (1998). Three-dimensional structure of the Stat3β homodimer bound to DNA. Nature.

[16] Park IH, Li C (2011). Characterization of molecular recognition of STAT3 SH2 domain inhibitors through molecular simulation. J. Mol. Recogn..

[17] Alotaibi BS (2023). New drug target identification in Vibrio vulnificus by subtractive genome analysis and their inhibitors through molecular docking and molecular dynamics simulations. Heliyon.

[18] Ajmal A (2023). Computer-assisted drug repurposing for thymidylate kinase drug target in monkeypox virus. Front. Cell. Infect. Microbiol..

[19] Daina A, Michielin O, Zoete V (2017). SwissADME: A free web tool to evaluate pharmacokinetics, drug-likeness and medicinal chemistry friendliness of small molecules. Sci. Rep..

[20] Ebrahimi KS (2021). In silico investigation on the inhibitory effect of fungal secondary metabolites on RNA dependent RNA polymerase of SARS-CoV-II: A docking and molecular dynamic simulation study. Comput. Biol. Med..

[21] Opo FADM (2021). Structure based pharmacophore modeling, virtual screening, molecular docking and ADMET approaches for identification of natural anti-cancer agents targeting XIAP protein. Sci. Rep..

[22] Tabti K (2023). Identification of a potential thiazole inhibitor against biofilms by 3D QSAR, molecular docking, DFT analysis, MM-PBSA binding energy calculations, and molecular dynamics simulation. Phys. Chem. Res..

[23] Zhou Y, Di B, Niu M-M (2019). Structure-based pharmacophore design and virtual screening for novel tubulin inhibitors with potential anticancer activity. Molecules.

[24] Manoharan S (2022). Screening of potent STAT3-SH2 domain inhibitors from JAK/STAT compound library through molecular dynamics simulation. Mol. Divers..

[25] Verma S (2016). Hydrophobic interactions are a key to MDM2 inhibition by polyphenols as revealed by molecular dynamics simulations and MM/PBSA free energy calculations. PLoS ONE.

[26] Erdem Koc G, Gokcimen A, Sahin F (2023). The effect of boric acid and sodium pentaborate pentahydrate-treated foreskin derived mesenchymal stem cells on liver fibrosis. Biol. Trace Elem. Res..

[27] Zhang L, Chan C (2010). Isolation and enrichment of rat mesenchymal stem cells (MSCs) and separation of single-colony derived MSCs. JoVE.

[28] Zhao J, Qi Y-F, Yu Y-R (2021). STAT3: A key regulator in liver fibrosis. Ann. Hepatol..

[29] Dong J (2021). Recent update on development of small-molecule STAT3 inhibitors for cancer therapy: From phosphorylation inhibition to protein degradation. J. Med. Chem..

[30] Chiba T (2016). STAT3 inhibitors for cancer therapy-the rationale and remained problems. EC cancer.

[31] Chen H (2023). Selectively targeting STAT3 using a small molecule inhibitor is a potential therapeutic strategy for pancreatic cancer. Clin. Cancer Res..

[32] Fagard R (2013). STAT3 inhibitors for cancer therapy: Have all roads been explored?. Jak-Stat.

