# Supplementary material for: Identification of novel STAT3 inhibitors for liver fibrosis, using pharmacophore-based virtual screening, molecular docking, and biomolecular dynamics simulations
Source: Sci Rep. 2023 Nov 17;13:20147. doi: 10.1038/s41598-023-46193-x (PMC10656421; doi:10.1038/s41598-023-46193-x)
Supplement: Supplementary file 1 — Supplementary Information. [file 41598_2023_46193_MOESM1_ESM.docx]

**Identification of novel STAT3 inhibitors for liver fibrosis, using Pharmacophore based virtual screening, molecular docking and biomolecular dynamics simulations**

**Supplementary Data:**

**RMSD Replica Graphs:**


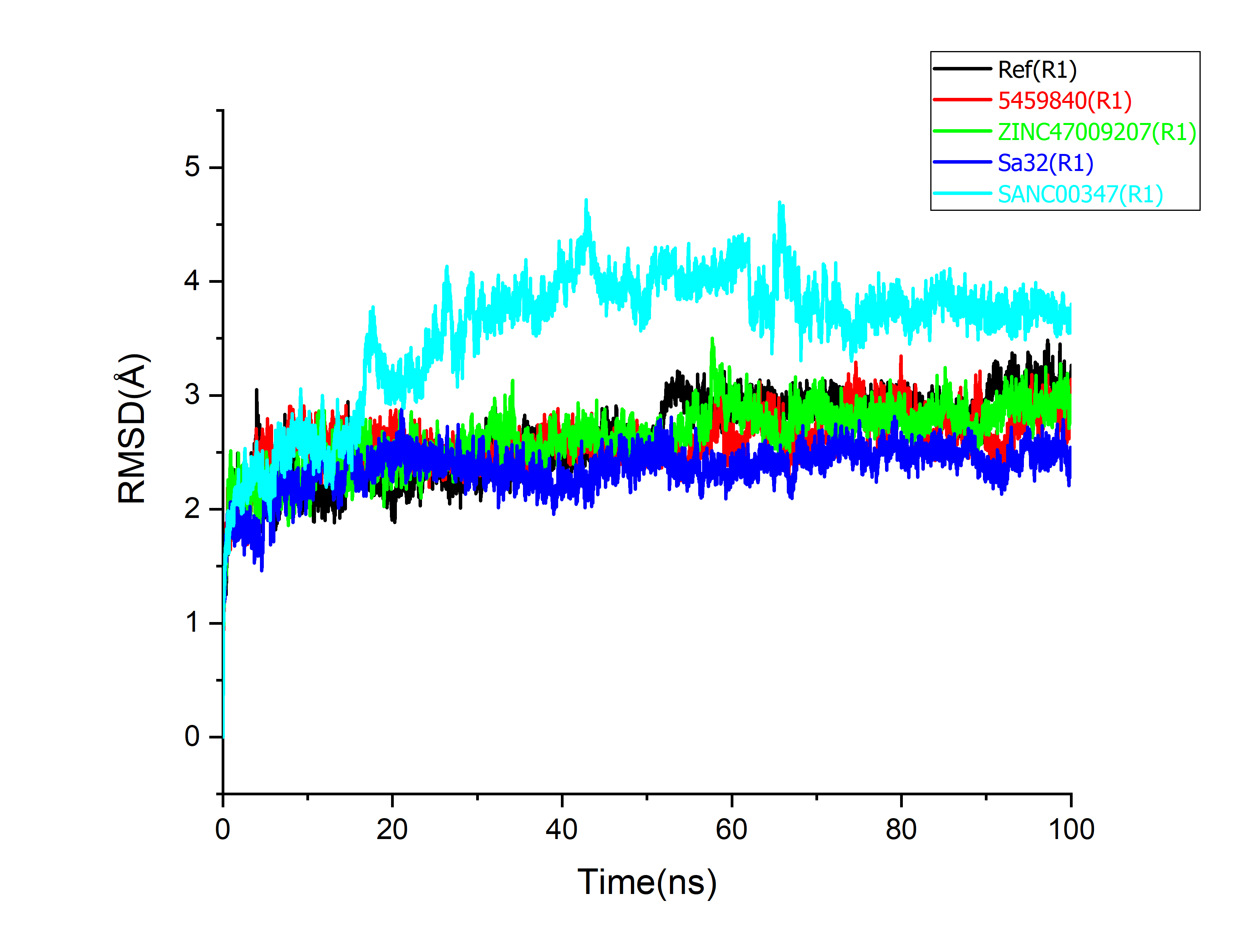


**Figure 4:** Root-mean square deviation (RMSD) analysis of Replica 1, black plot in graph shows the reference complex and Red, Green, Blue and Sky blue plotted indicates 5459840, ZINC47009207, Sa32 and SANC00347 respectively.


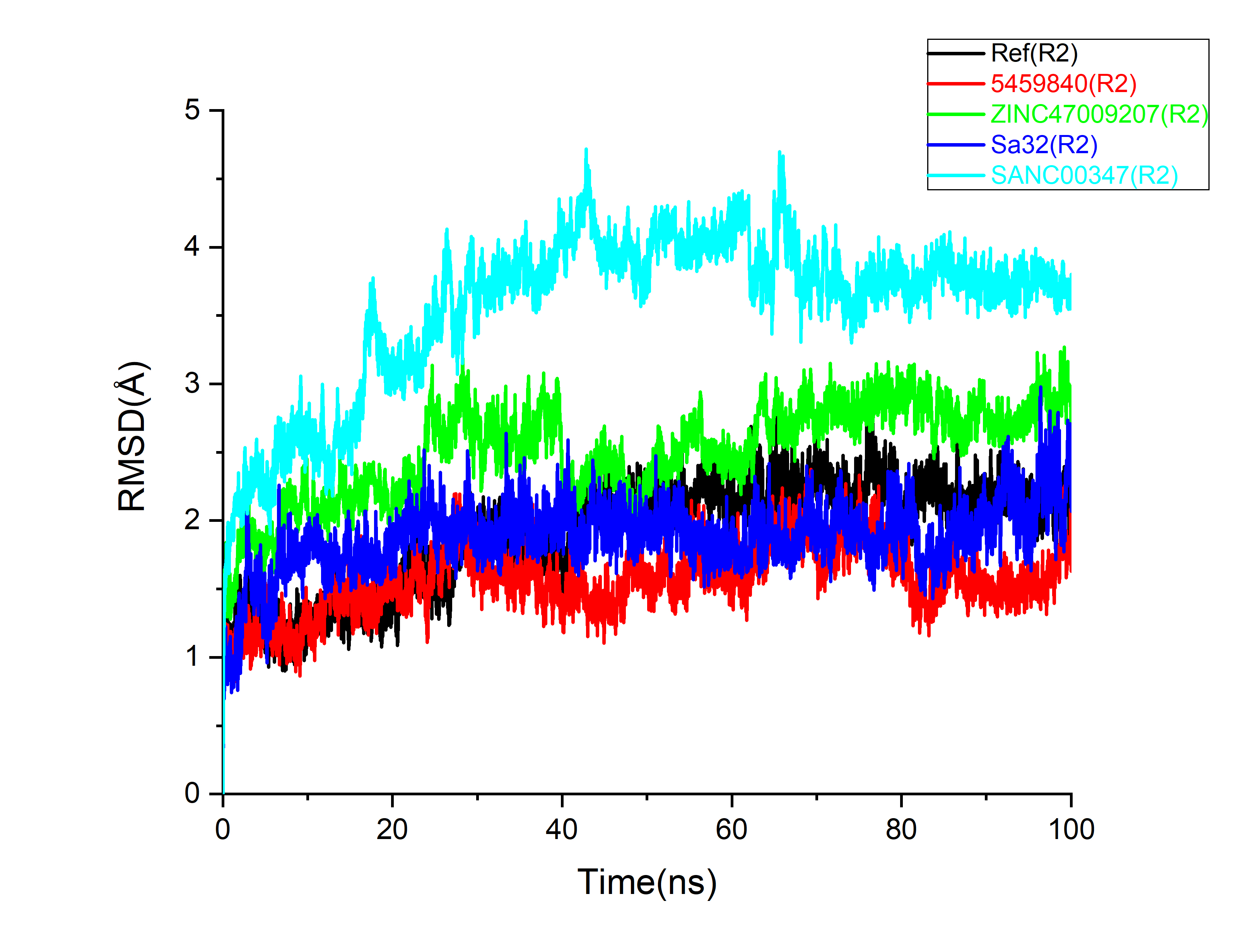


**Figure 4s:** Root-mean square deviation (RMSD) analysis of Replica 2, black plot in graph shows the reference complex and Red, Green, Blue and Sky blue plotted indicates 5459840, ZINC47009207, Sa32 and SANC00347 respectively.

**RMSF Replicas Graphs:**

**Figure 5:** Root-mean-square fluctuation analysis of replica 1, the black line in graph represents reference complex. And the rest of Red, Green, Blue and Sky blue indicate 5459840, ZINC47009207, Sa32 and SANC00347 respectively.


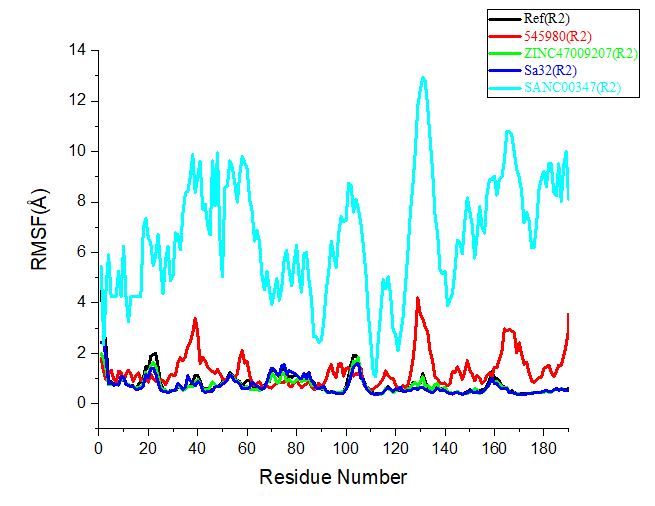


**Figure 5s:** Root-mean-square fluctuation analysis of replica 2, the black line in graph represents reference complex. And the rest of Red, Green, Blue and Sky blue indicate 5459840, ZINC47009207, Sa32 and SANC00347 respectively.

**Rg Replica Graphs:**

**Figure 6:** Rg analysis of replica 1, Black, Red, green, blue and sky-blue shows Ref, 5459840, ZINC47009207, Sa32 and SANC00347 respectively.


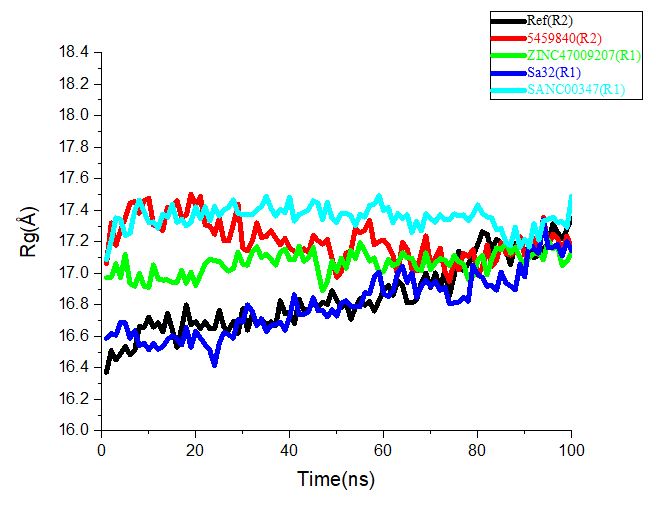


**Figure 6s:** Rg analysis of replica 2, Black, Red, green, blue and sky-blue shows Ref, 5459840, ZINC47009207, Sa32 and SANC00347 respectively.
